# Time Difference of Arrival (TDoA) Localization Combining Weighted Least Squares and Firefly Algorithm

**DOI:** 10.3390/s19112554

**Published:** 2019-06-04

**Authors:** Peng Wu, Shaojing Su, Zhen Zuo, Xiaojun Guo, Bei Sun, Xudong Wen

**Affiliations:** College of Intelligence Science and Technology, National University of Defense Technology, Changsha 410073, China; pengwu9510@163.com (P.W.); susj-5@163.com (S.S.); jeanakin@nudt.edu.cn (X.G.); beys1990@163.com (B.S.); wenxudong13@163.com (X.W.)

**Keywords:** TDoA, weighted least squares, firefly algorithm, hybrid-FA

## Abstract

Time difference of arrival (TDoA) based on a group of sensor nodes with known locations has been widely used to locate targets. Two-step weighted least squares (TSWLS), constrained weighted least squares (CWLS), and Newton–Raphson (NR) iteration are commonly used passive location methods, among which the initial position is needed and the complexity is high. This paper proposes a hybrid firefly algorithm (hybrid-FA) method, combining the weighted least squares (WLS) algorithm and FA, which can reduce computation as well as achieve high accuracy. The WLS algorithm is performed first, the result of which is used to restrict the search region for the FA method. Simulations showed that the hybrid-FA method required far fewer iterations than the FA method alone to achieve the same accuracy. Additionally, two experiments were conducted to compare the results of hybrid-FA with other methods. The findings indicated that the root-mean-square error (RMSE) and mean distance error of the hybrid-FA method were lower than that of the NR, TSWLS, and genetic algorithm (GA). On the whole, the hybrid-FA outperformed the NR, TSWLS, and GA for TDoA measurement.

## 1. Introduction

Target localization based on a group of sensor nodes whose positions are known has been extensively studied in research on signal processing [1,2,3]. It has been applied widely in military and civil fields, including sensor networks [4], wireless communication [2], radar [5], navigation, and so forth [6,7,8]. Commonly adopted positioning methods include the signal’s time of arrival (ToA) [9], time difference of arrival (TDoA), frequency difference of arrival (FDoA) [10,11,12,13], or doppler shift [14]. Compared with FDoA, the TDoA and ToA methods can achieve higher positioning accuracy and require only one channel for each sensor node to perform the measurement, which can minimize the load requirement for a single-sensor node.

For a passive location system based on TDoA, once the measured data are obtained, the range difference between the target and two different sensor nodes can be calculated. In this connection, a set of hyperbolic equations or hyperboloids can be obtained and the solution of the equations is the coordinate of the target. Generally, the solving algorithms commonly adopted include iterative, analytical, and search methods.

The procedure for solving equations from the TDoA method is complex and difficult because the equations are nonlinear, and many studies have been carried out on how to solve this issue. The main idea of the Taylor series method is to expand the first Taylor series of the nonlinear positioning equation at the initial estimation of the target position and then solve the equations by iteration [15]. The advantage of this method is that it can fuse multiple observation data. Yang et al. transformed the equation into a constrained weighted least squares (CWLS) estimation problem by introducing auxiliary variables, and then the Newton iteration method was adopted to solve the problem [16]. In [17], nonconvex TDoA localization was transformed into a convex semidefinite programming (SDP) problem, and the approximate result was taken as the initial value for the Newton iteration method. All of these methods are iterative. Compared with iterative methods, the closed-form method does not need the initial estimation of the target’s location and iterative solving is not necessary either. For example, Chan and Ho [18] transformed nonlinear equations into pseudolinear equations by introducing auxiliary variables. Then, the equations were solved by two-step weighted least squares (TSWLS). One downside of this algorithm is that the result is substantially different than the actual position when the signal-to-noise ratio (SNR) is low. Considering this problem, the constrained total least squares (CTLS) method was proposed in [19,20,21]. While it is not a closed-form method and Newton iterations are needed, the complexity of the CTLS method is much higher than that of the TSWLS method. The approximate maximum likelihood (AML) method [22] was proposed, which can obtain a linear equation from the maximum likelihood function and then the target location can be calculated. The AML method has better positioning performance than the TSWLS method.

In addition to the traditional TSWLS, iteration methods, and so on, many scholars have investigated new methods to enhance positioning accuracy. Two new shrinking-circle methods were proposed (SC-1 and SC-2) to solve a TDoA-based localization problem in a 2-D space [23]. Additionally, a weighted least squares (WLS) algorithm with the cone tangent plane constraint for hyperbolic positioning was proposed, which added the distance between the target and the reference sensor as a new dimension [24]. The theoretical bias of maximum likelihood estimation (MLE) is derived when sensor location errors and positioning measurement noise both exist [25]. Using a rough estimated result by MLE to subtract the theoretical bias can deliver a more accurate source location estimation. Apart from this, research based on certain typical algorithms has been carried out to extract and calculate the TDoA of ultrahigh frequency (UHF) signals [26]. The AML algorithm was proposed for determining a moving target’s position and velocity by utilizing TDoA and FDoA measurements [27].

It is also efficient to use a search algorithm to calculate the position of a target. A hybrid genetic algorithm (GA) was proposed to enhance solution accuracy [18]. Nature-inspired algorithms are powerful algorithms for optimization. The firefly algorithm (FA) is one such nature-inspired algorithm, which was proposed in 2008. Using the FA for multimodal optimization applications with high efficiency has been proposed [28,29].

In general, among the methods for solving TDoA equations, analytical and iterative methods both have limitations. Research on algorithms that are robust and have low computational complexity is still worthy of study. Search algorithms for TDoA measurements can provide accurate results, although the efficiency will inevitably decrease when there are many estimated parameters [29]. Therefore, it is necessary to develop a highly efficient search algorithm for TDoA.

This paper is organized as follows: Section 2 introduces the basic model of TDoA measurement. Section 3 formulates the basic principle of WLS. Section 4 provides the main steps of the FA. Section 5 details the hybrid-FA methods proposed in this paper. Section 6 presents the results of simulations and experiments to support the theoretical analysis.

## 2. Problem Description

In this section, 2-D target localization based on TDoA measurement is presented in the line-of-sight environment. Assume that there are *N* (*N* ≥ 3) sensor nodes, which can also be called basic sensors (*BSs*), to determine the position of the target. The coordinates of the sensor nodes are known, which are $s_{i}=(a_{i},b_{i}{)}^{T},i\in \left\{1,2,...,N\right\}$, where ${\left[\cdot \right]}^{T}$ denotes the matrix transpose. Assume that the target’s coordinate is $p=(x,y{)}^{T}$.

**Figure 1:**
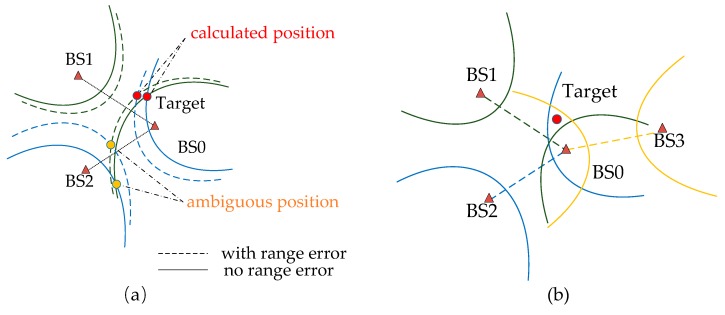
The principle of time difference of arrival (TDoA) measurement. (**a**) The diagram when there are three basic sensors of TDoA. (**b**) Multiple hyperbolas for the optimal position.

As shown in Figure 1a, there are three basic sensors in the 2-D plane to determine the position of the target which form two groups of hyperbolas [24]. The hyperbola has two intersections in the absence of noise and there is one ambiguous position in them. When noise exists, the other two groups of hyperbolas have the other two intersections and both intersections have errors. In order to avoid the ambiguous position, it is advisable to increase the number of the sensors. As demonstrated in Figure 1b, the four basic sensors form three groups of hyperbolas and there is only one intersection without noise, which is the estimated position of the target. When noise exists, it is necessary to follow certain principles to obtain the optimal results.

Take the first basic sensor $BS_{1}$ as a reference sensor and assume that the signal propagates in a straight line between the target and each basic sensor without considering the influence of non-line-of-sight propagation. Assume that the times when the signal arrives at basic sensors $BS_{1}$ and $BS_{i}$ are $t_{1}$ and $t_{i}$, respectively, and the propagation speed of the signal is c. The range of difference between the target and two basic sensors $BS_{1}$ and $BS_{i}$ is $\left\{r_{i,1}\right\}$. This paper assumes that range difference errors $\left\{n_{i}\right\}$ are independent Gaussian random variables with zero mean and known variance $\sigma_{i}^{2}$, i.e., $N(0,\sigma_{i}^{2})$. We can obtain

(1) $$r_{i,1}=c|t_{1}-t_{i}|$$

(2) $$r_{i,1}=d_{i,1}+n_{i,1}{}_{}{,}_{}i\in \left\{2,...,N\right\}.$$

Thus,

(3) $${c|t}_{1}{-t}_{i}{|=d}_{i,1}{+n}_{i,1}$$

where $d_{i,1}{=d}_{i}-d_{1}$. Here, distances between the target and the receiver pair $BS_{1}$ and $BS_{i}$ can be expressed as follows:

(4) $$d_{1}=\sqrt{({x-a}_{1}{)}^{2}+({y-b}_{1}{)}^{2}}$$

(5) $$d_{i}=\sqrt{({x-a}_{i}{)}^{2}+({y-b}_{i}{)}^{2}},\;i\in \left\{2,...,N\right\}.$$

Actually, the process of obtaining results based on TDoA measurements is the process of solving the *N* − 1 equations as shown in Equation (3) and obtaining the optimal solution.

## 3. WLS Method 

Usually, there are iterative methods, such as those mentioned in Section 1, to solve the equations, for which the computational burden is heavy. In this section, the WLS method is introduced based on TDoA measurements [29]. The sum of squares of residuals is defined as $J_{NLS}(\tilde{x})$:

(6) $$J_{NLS}(\tilde{x})=min\sum_{i=1}^{N}R_{i}^{2}(\tilde{x})$$

where $\tilde{x}$ represents the optimization variable, and residual $R_{i}^{}(\tilde{x})$ can be expressed as

(7) $$R_{i}^{}(\tilde{x}){=\tilde{r}}_{i,1}{-r}_{i,1}$$

where ${\tilde{r}}_{i,1}$ is the measured value. Therefore, the optimal solution $\hat{p}$ according to the principle of minimum variance is

(8) $$\hat{p}=arg\underset{\tilde{x}\in R^{2}}{min}J_{NLS}(\tilde{x}).$$

Nonlinear hyperbolic equations can be transformed as follows:

(9) $$r_{i,1}+\sqrt{({x-a}_{1}{)}^{2}+({y-b}_{1}{)}^{2}}=\sqrt{({x-a}_{i}{)}^{2}+({y-b}_{i}{)}^{2}}+n_{i,1},\;i\in \left\{2,...,N\right\}.$$

After mathematical transformation, we can obtain

(10) $$({x-a}_{1})(a_{i}{-a}_{1})+({y-b}_{1})(b_{i}{-b}_{1}){+r}_{i,1}d_{1}=\frac{1}{2}\left[{\left(a_{i}{-a}_{1}\right)}^{2}+{\left(b_{i}{-b}_{1}\right)}^{2}{-r}_{i,1}{}^{2}\right]{+d}_{i}n_{i,1},i\in \left\{2,...,N\right\}$$

where the second-order term $n_{i,1}{}^{2}$ is ignored and $e_{i,1}{=d}_{i}n_{i,1}$. We can obtain

(11) AX=θ+e

in which

(12) $$A=\left[\begin{array}{ccc} a_{2}-a_{1} & {\;b}_{2}-b_{1} & {\;r}_{2,1}\\ a_{3}-a_{1} & {\;b}_{3}-b_{1} & {\;r}_{3,1}\\ \vdots & \vdots & \vdots \\ a_{N}-a_{1} & {\;b}_{N}-b_{1} & {\;r}_{N,1} \end{array}\right]$$

(13) $$X=\left[x-a_{1}\;y-b_{1}\;d_{1}\right]$$

(14) $$\theta =\frac{1}{2}\left[\begin{array}{c} (a_{2}-a_{1}{)}^{2}+(b_{2}-b_{1}{)}^{2}{-r}_{2,1}^{2}\\ (a_{3}-a_{1}{)}^{2}+(b_{3}-b_{1}{)}^{2}{-r}_{3,1}^{2}\\ \vdots \\ (a_{N}-a_{1}{)}^{2}+(b_{N}-b_{1}{)}^{2}{-r}_{N,1}^{2} \end{array}\right],$$

(15) $$e={\left[e_{2,1}{\;e}_{3,1}\cdots {\;e}_{N,1}\right]}^{T}.$$

Then, the WLS objective function can be expressed as

(16) $$J_{WLS}(X)=(AX-\theta {)}^{T}W(AX-\theta )$$

where the weighting matrix is $W={\left(E\left\{ee^{T}\right\}\right)}^{-1}$.

Thus,

(17) $${\hat{x}}_{WLS}=(A^{T}WA{)}^{-1}A^{T}W\theta .$$

The WLS method is often adopted because of its simplicity and lower computational burden. As the equations are approximated in the process of simplification, it has low accuracy.

## 4. Firefly Algorithm

In this section, the principle of the FA is introduced, which is a kind of heuristic algorithm inspired by the flickering behavior of fireflies. The three following idealized rules are needed for the model of this algorithm [30]:

- Each firefly will be attracted by the other fireflies regardless of their sex.
- The higher the brightness of the firefly, the greater the attractiveness of the firefly. In this connection, a less bright firefly will move towards a brighter one.
- The brightness of the fireflies is associated with the objective function.

Through the attraction between the brighter and less bright fireflies, the fireflies will eventually gather around the brightest firefly, which can realize the optimization of the objective function. We can use FA search methods to obtain the optimal result that satisfies Formula (18):

(18) $$J_{NLS}(\tilde{x})=min\sum_{i=1}^{N}R_{i}^{2}(\tilde{x}).$$

In the search space, fireflies move towards brighter fireflies continuously to complete optimization until the preset termination condition of the algorithm is reached.

Assuming that the number of fireflies is *N* and the dimension is *D*, the positions of the $i^{\mathrm{th}}$ and $j^{\mathrm{th}}$ fireflies are $x_{i}=(x_{i1}{,x}_{i2},\cdots {,x}_{\mathrm{iD}}),i=1,2,\cdots ,N$ and $x_{j}=(x_{j1}{,x}_{j2},\cdots {,x}_{\mathrm{jD}}),j=1,2,\cdots ,N$. $r_{\mathrm{ij}}$ is the distance between the $i^{\mathrm{th}}$ and $j^{\mathrm{th}}$ fireflies, which can be calculated as follows:

(19) $$r_{ij}=\|x_{i}{-x}_{j}\|=\sqrt{\sum_{d=1}^{D}(x_{id}{-x}_{jd}{)}^{2}}.$$

Among them, $x_{\mathrm{id}}$ and $x_{jd}$ represent the positions of the $i^{\mathrm{th}}$ and $j^{\mathrm{th}}$ fireflies, respectively.

The relative brightness of the fireflies is defined as

(20) $${I=I}_{0}e^{-\gamma r_{\mathrm{ij}}{}^{2}}$$

where $I_{0}$ represents the brightness of the firefly, which is proportional to the value of the objective function. γ is the coefficient of absorbing light intensity, which is usually defined as a constant. $r_{\mathrm{ij}}$ denotes the distance of fireflies *i* and *j*.

The attractiveness of the fireflies is defined as follows:

(21) $$\beta =\beta_{0}e^{-\gamma r_{\mathrm{ij}}{}^{2}}$$

where $\beta_{0}$ denotes the factor of maximum attraction degree, indicting the attractiveness of the position with the maximum brightness. From Formula (21), we understand that attractiveness decreases with the increase of the distance and the coefficient of absorbing light intensity.

The update of the location is

(22) $$x_{\mathrm{id}}(t+1){=x}_{\mathrm{id}}(t_{})+\beta \left(x_{\mathrm{jd}}(t_{})-x_{\mathrm{id}}(t_{})\right)+\varepsilon \cdot \alpha_{i}(t_{})$$

where $x_{\mathrm{id}}(t_{})$ and $x_{\mathrm{jd}}(t_{})$ are the positions of the $i^{\mathrm{th}}$ and $j^{\mathrm{th}}$ fireflies after the $t^{\mathrm{th}}$ generation. $\alpha_{i}(t_{})$ denotes the step factor of the $t^{\mathrm{th}}$ generation. The range of the value ε is [−0.5, 0.5], which sequences with uniform distribution.

The process of optimization is as follows. Fireflies with varying degrees of brightness are randomly dispersed in the solution space. The brightness and attractiveness of the fireflies can be calculated according to Equations (20) and (21), respectively. The less bright fireflies will move towards the brighter one. In order to avoid falling into the local optimum, the perturbation term $\varepsilon \cdot \alpha_{i}(t_{})$ is added to the process of location updating. Finally, the fireflies will gather around the firefly with highest brightness. The optimal result can thus be obtained. The flowchart for this can be found below.

**Step 1.** Initialize the parameters in the algorithm. Set the number of fireflies *N*, the factor of maximum attraction degree $\beta_{0}$, and maximum iteration number or convergence criterion.

**Step 2.** Initialize the location of the fireflies randomly and calculate the value of the objective function as the original brightness.

**Step 3.** Calculate the brightness and attractiveness of fireflies referring to Equations (20) and (21), respectively, and determine the moving direction of the fireflies according to their relative brightness.

**Step 4.** Update the location of the fireflies according to Equation (22) and add the perturbation terms.

**Step 5.** Recalculate the brightness of the fireflies after updating the location of the fireflies.

**Step 6.** When the convergence criterion is satisfied or the maximum number of iterations reached, go to the next step; otherwise, go to Step 3.

**Step 7.** Output the global extremum and optimal value.

## 5. Hybrid-FA Method

While it usually takes more time to use a search algorithm than iterative methods, this method provides higher accuracy and thus has great potential in practical applications. There are certain reasons for the longer solution time. One significant cause is that it will search the optimal result in the global scope. In this context, if some reasonable regional restrictions are given, a search algorithm, including the FA method, will reduce the computation amount as well as ensure the accuracy of the result.

In this study, the WLS and FA methods were combined for optimal implementation. Since it is easy to obtain the initial result by the WLS method, the initial result can be used to provide the limited area for the FA method.

Assume that the search area is square and the length of a side is l. If the result obtained by the WLS method is $\left(x_{\mathrm{wls}},y_{\mathrm{wls}}\right)$, which is the initial value, then the constrained region can be given for the FA method as $\left[x_{\mathrm{wls}}\pm \frac{1}{4}l]\times [y_{\mathrm{wls}}\pm \frac{1}{4}l\right]$ to ensure the target falls into the restricted region as much as possible. Additionally, new firefly positions out of the restricted region are ignored in this method.

**Figure 2:**
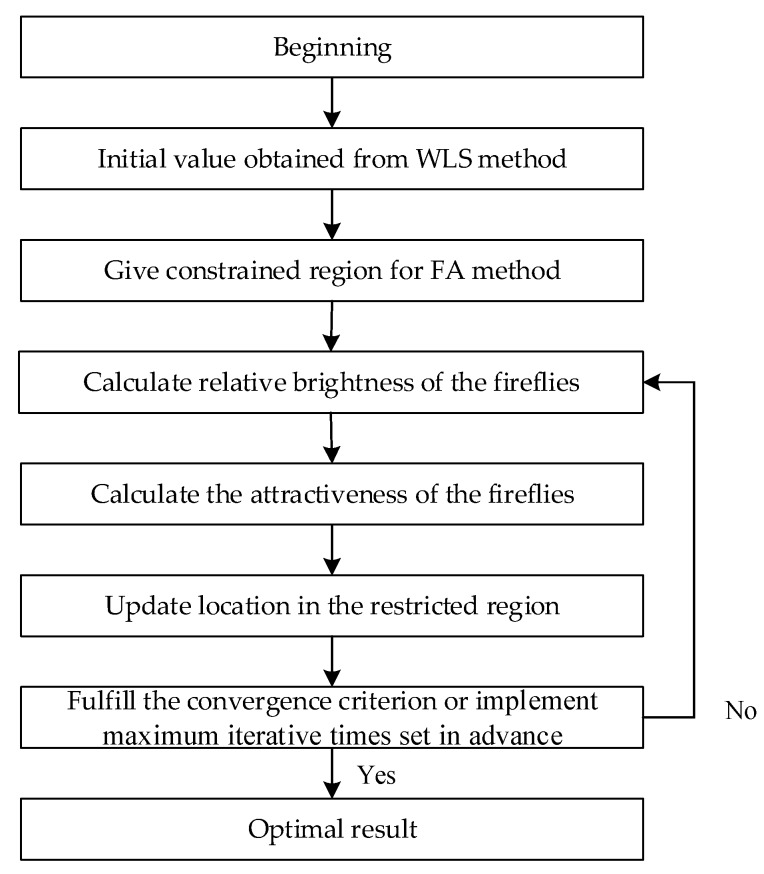
The diagram of hybrid firefly algorithm (hybrid-FA) method.

As shown in Figure 2, the WLS and FA methods are combined for optimal implementation. The initial value is obtained by the WLS method, then the initial result can be used to provide the restrained area for the FA method to search for the optimal result. There are two conditions when the algorithm ends, fulfilling the convergence criterion or implementing maximum iterative times set in advance. As for the former condition, the values of the objective function obtained in the ${i-1}^{\mathrm{th}}$ and $i^{\mathrm{th}}$ iteration are compared. Assuming that the objective function is $F^{}$, then the ending condition can be expressed as follows:

(23) $$\|X^{}(i^{\mathrm{th}}){-X}^{}({i-1}^{\mathrm{th}})\|\leq \varepsilon_{1}$$

(24) $$\|F^{}(i^{\mathrm{th}})-F^{}({i-1}^{\mathrm{th}})\|\leq \varepsilon_{2}$$

where $\varepsilon_{1}$ and $\varepsilon_{2}$ are positive predetermined numbers.

## 6. Results 

### 6.1. Preprocessing

Simulations and indoor experiments were conducted and the results of the proposed method and other commonly used methods are presented and compared here. Before the simulations and experiments, the definition of the SNR and the evaluation index are given.

In this paper, the SNR of the signal is defined as

(25) $$SNR=10\log\frac{d_{i,1}}{\sigma_{i}{}^{2}}\;\mathrm{dB}$$

where $d_{i,1}$ denotes the distance difference between the target and the basic sensors $BS_{1}$ and $BS_{i}$, and $\sigma_{i}$ represents the standard deviation of the noise.

Therefore, when used in practice, if the SNR is known in advance, the variance of noise can be obtained:

(26) $$\sigma_{i}{}^{2}=\frac{d_{i,1}{}^{2}}{10^{SNR/10}}.$$

Otherwise, when the SNR is not known in advance, the variance of noise can be obtained using the approximate method. The performance index of the receiver is usually known according to the specifications or can be measured by testing. Here, the signal’s time of arrival was measured by the receiver. Assume that the measurement error targeting at the time of arrival of different receivers is no more than $\Delta {\hat{t}}_{i}{}_{}\left(i=1,2,...,M\right)$, where M denotes the number of the receivers. Assume that the time difference of arrival between *BS*_1_ and *BS*_i_ is $\Delta t_{1,i}$.

Then, we can get

(27) $$\Delta t_{1,i}=\left|t_{1}-t_{i}\right|$$

where $t_{1}$ and $t_{i}$ denote the measured values when the signal arrives at the basic sensors $BS_{1}$ and $BS_{i}$, respectively.

The variance of the noise of $\Delta t_{1,i}$ can be approximated to

(28) $$\sigma_{i}{}^{2}=c^{2}\left(\Delta {\hat{t}}_{1}{}^{2}+\Delta {\hat{t}}_{i}{}^{2}\right)$$

where c represents the propagation velocity of the signal.

The localization performance was evaluated referring to the root-mean-square error (RMSE), which is defined as

(29) $$RMSE=\sqrt{\frac{1}{n}\sum_{i=1}^{n}\left[({\hat{x}}_{i}-x{)}^{2}+({\hat{y}}_{i}-y{)}^{2}\right]}$$

where n represents the number of simulation times, (x,y) are the real position coordinates of the target, and $\left({\hat{x}}_{i},{\hat{y}}_{i}\right)$ are the estimated positions based on the $i^{\mathrm{th}}$ calculation. RMSE was used to measure the average coordinate distance between the estimated target position and the actual target position. The lower it is, the higher the accuracy is.

### 6.2. Simulation Conditions

**Figure 3:**
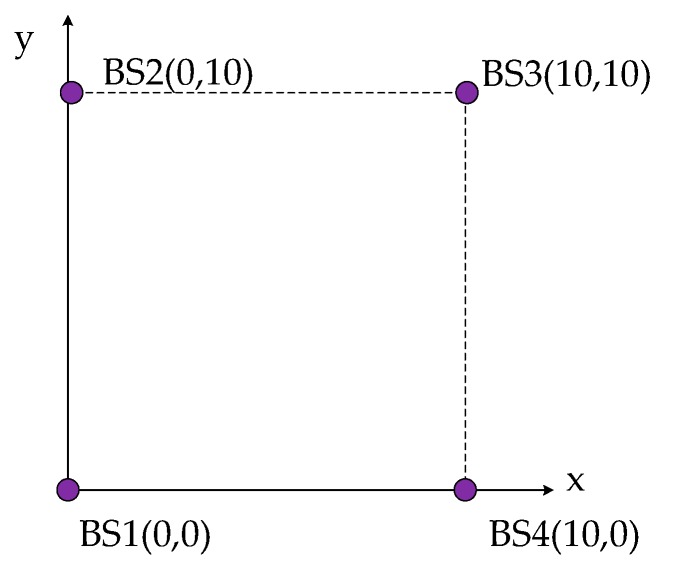
The layout of four sensor nodes in the simulation experiment.

The simulation was performed using Matlab 2014a and all the results were obtained on the same computer with a 1.8 GHz CPU and 8 G RAM. Assume that the coordinates of the four basic sensors were *BS1*(0,0), *BS2*(0,10), *BS3*(10,10), and *BS4*(10,0), where *BS1* is the reference basic sensor. The layout of the four sensor nodes is shown in Figure 3.

### 6.3. The Robustness 

The results of the simulations were as follows when the SNR = 30 dB. In order to have a better display, the vertical coordinate represents -RMSE. Therefore, the optimal result was the coordinate when the -RMSE reached the maximum.

**Figure 4:**
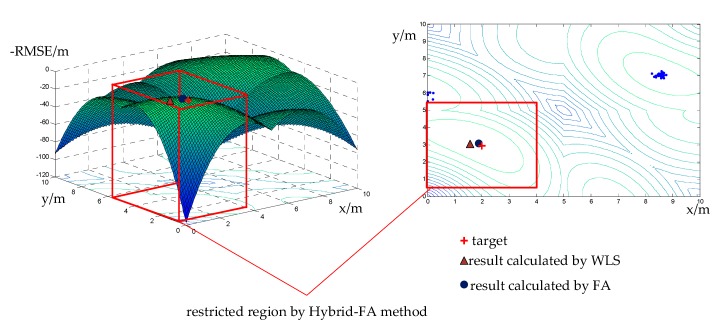
The diagram of hybrid-FA and FA.

As illustrated in Figure 4, the FA method searched for the optimal result in the global region ([0,10] × [0,10]). From the figure, we can see that the result obtained by the FA method was even closer to the actual target location than that obtained by the WLS method, while the hybrid-FA method simply searched in the square marked in red. In this connection, it reduced the computational burden and maintained high accuracy.

Table 1 shows the comparison between FA and hybrid-FA under the same condition when SNR = 30 dB. When the RMSE reached 0.03741 m, the hybrid-FA method only needed to iterate 30 times, while 100 times was required for the FA method by itself. The simulation results demonstrate the efficiency of the hybrid-FA method.

**Figure 5:**
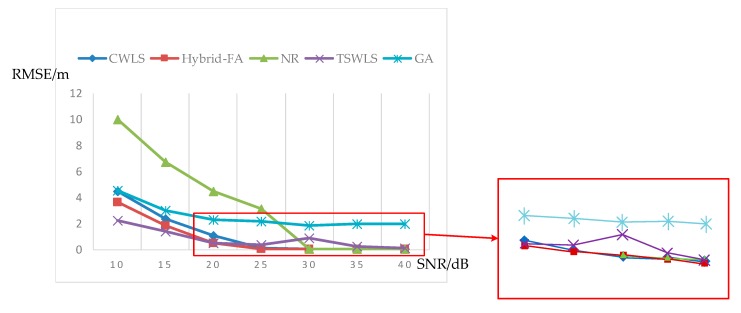
The comparison of the four algorithms for TDoA measurement.

In this section, the results of the commonly used algorithms CWLS [31], Newton–Raphson (NR) [29], TSWLS [18], and GA [30], were compared with the hybrid-FA. The GA method is a search algorithm that is commonly used for optimization. For these methods, the number of iterations was 30 and the coordinate of the target was set as (2,3). Figure 5 illustrates the compared results.

As demonstrated in Figure 5, with the increase of SNR, the RMSE of each algorithm tended to decrease, which means the accuracy of the position had improved. When the SNR = 10 and 15 dB, the RMSE of TSWLS was the lowest, while the RMSE of the other five methods was typically higher than 2 m. It was hard to achieve high accuracy when the SNR was very low, as the acquired data was limited. The data of FDoA or TOA should be combined to get higher accuracy. When the SNR = 20, 25, 35, and 40 dB, the RMSE of the hybrid-FA was the lowest, which was 0.46077, 0.03788, 0.02427, and 0.0177 m, respectively. When SNR = 30 dB, the RMSE of the CWLS method was 0.0001 m lower than that of hybrid-FA method. On the whole, the hybrid-FA method was better than the NR, TSWLS, and GA methods when the SNR ranged from 20 to 40 dB in the simulations. The result of GA is also shown in Figure 5. The RMSE of GA was higher than that of the proposed scheme, which illustrates that the proposed scheme is more appropriate for optimization than GA.

### 6.4. Experiment

Experiments were carried out to verify the rationality of the algorithms [23,32]. The coordinates of four speakers were *BS1*(0,0)m, *BS2*(0,10)m, *BS3*(10,10)m, and *BS4*(10,0)m, which were used to generate signals. The speakers emitted chirp signals, which were continuous impulse signals of 2.5 kHz. A phone was placed at the same height to receive the sound signal as well as record the receiving time. In this experiment, time-division multiplexing was adopted. The emitting cycle was 1 s, and the speakers emitted 100 ms long signals one after another. The speaker of *BS1* emitted 100 ms long signals at the beginning of every emitting cycle. The speakers of *BS2*, *BS3*, and *BS4* emitted 100 ms long signals at the 250th, 500th, and 750th ms, respectively. Take the speaker of *BS1* as a reference speaker. The receiving time data were saved to a text file and exported to the computer to be processed in Matlab 2014a.

Assume that $t_{i}^{j}$ represents the time when the phone receives the signal from the *BSi* speaker at the jth emitting cycle. The time difference of arrival between *BS1* and *BSi* can be expressed as

(30) $$\Delta t=\left|t_{i}^{j}-t_{1}^{j}-\frac{i-1}{4}T\right|$$

where *T* denotes the emitting cycle. Thus, the range of difference between the receiver and speakers *BS1* and *BSi* can be expressed as

(31) $$r_{i,1}^{o}=c^{o}\Delta t$$

where $c^{o}$ denotes the propagation velocity of the signal. Then, the equations can be obtained and solved by the localization algorithms.

**Figure 6:**
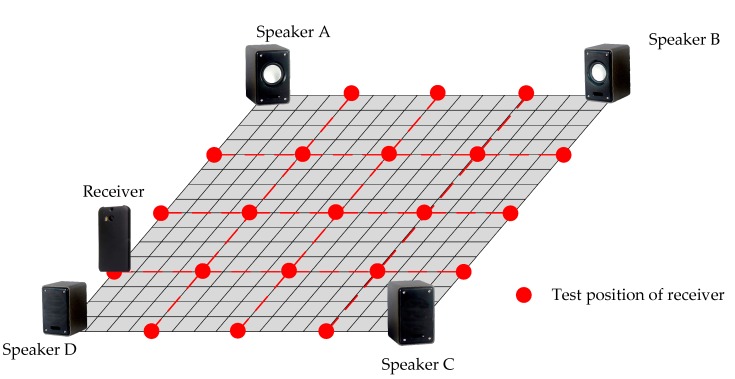
The sketch of the first experiment.

Firstly, the experiment was conducted to verify the performance of the five localization methods. There were 19 test positions of the receiver and the distribution of receiver occupied the search region as much as possible. At each test position, 50 trials were conducted under the same conditions. In total, 950 trials were carried out in this experiment. The sketch of the experiment is shown in Figure 6.

In Figure 6, the red points represent the position of the receiver and the distance of the two adjacent test points is 2.5 m. For the results, the trials with RMSE greater than 2.5 m were considered as bad results. The result of RMSE is the mean of the results from all trials for each method.

**Figure 7:**
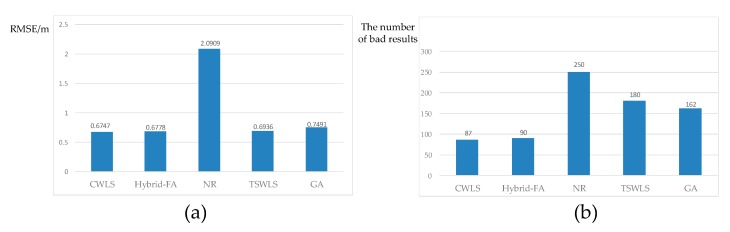
The results of the first experiment. (**a**) The RMSE of the five methods. (**b**) The number of bad results from the five methods.

The RMSE of the five methods in this experiment and the amount of bad results are shown in Figure 7. The RMSE of the hybrid-FA method was 0.6778 m, which was lower than that of NR, TSWLS, and GA and 0.0031 m higher than that of CWLS. As for the amount of bad results, for the hybrid-FA method, it was 90, which was less than that of the NR, TSWLS, and GA methods and 3 more than that of CWLS. It can be concluded that the performance of the hybrid-FA method was superior to that of the NR, TSWLS, and GA methods for TDoA measurement.

**Figure 8:**
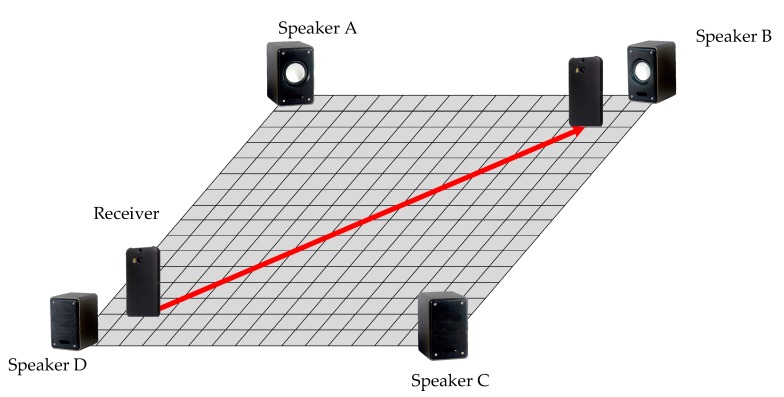
The setting of second experiment.

In the experiment, the phone moved along the red path slowly, as shown in Figure 8. The A series of data was recorded and the results were calculated according to the different methods. The amount of the test position was 19 in the experiment. The discrete position sequence was obtained, then the Kalman filter with the same parameters was used to smooth the motion trail. The final results are shown below.

**Figure 9:**
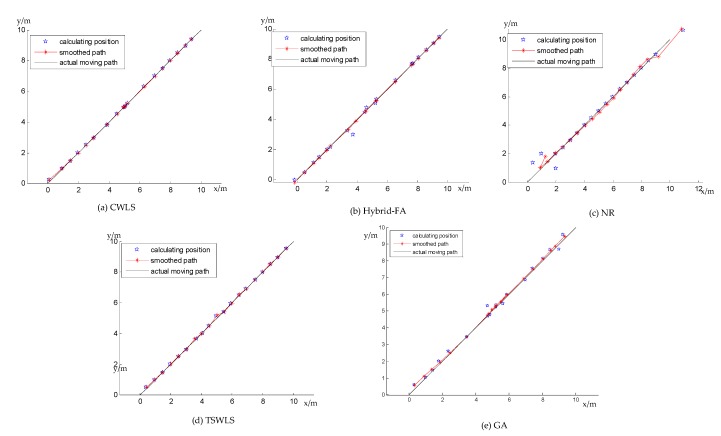
The trajectory tracking of the five methods based on TDoA. (**a**) The trajectory tracking of constrained weighted least squares (CWLS). (**b**) The trajectory tracking of hybrid-FA. (**c**) The trajectory tracking of Newton–Raphson (NR). (**d**) The trajectory tracking of two-step weighted least squares (TSWLS). (**e**) The trajectory tracking of the genetic algorithm (GA).

Figure 9 illustrates the trajectory tracking of the CWLS, hybrid-FA, NR, TSWLS, and GA methods. In Figure 9, the blue point is the discrete position solved by localization algorithms, the black line is the actual trajectory of the target, the red point is the estimated position obtained by smoothing the discrete position sequence using the Kalman filter, and the red line is the smoothed trajectory of the target. It was difficult to arrive at a conclusion solely through observation. For this reason, the mean distance error was introduced to compare the performances of the different methods. Assume that the coordinate of the smoothed position is $\left(x_{i}^{o},y_{i}^{o}\right)$ for each method and $d_{i}^{o}$ is the distance between the smoothed position and the line y = x in the coordinate system, which is the actual moving path of the receiver. Thus, the mean distance error is defined as follows.

(32) $$\mathrm{mean}\;\mathrm{distance}\;\mathrm{error}\;=\;\frac{1}{N}\sum_{i=1}^{N}d_{i}^{o}$$

Table 2 shows the mean distance error of the CWLS, hybrid-FA, NR, TSWLS, and GA methods. The mean distance error of the hybrid-FA method in this experiment was 0.03419 m, which was less than that of the NR, TSWLS, and GA methods and 0.000985 m more than that of CWLS. It can be concluded that the hybrid-FA method outperformed the NR, TSWLS, and GA methods for TDoA measurement.

## 7. Conclusions

For TDoA measurement, a good algorithm should balance calculation and precision. In this paper, a hybrid-FA method was proposed that combined the WLS and FA methods, which used the result from WLS with low computational burden to provide a reasonable limit to the search region for the FA method. The results of the proposed method were compared with the CWLS, NR, TSWLS, and GA methods using simulations and two experiments, which demonstrated the validity and limitations of the proposed method.

As expected, the hybrid-FA method could cut down the computation of the algorithm with high accuracy compared with using the FA only. Additionally, the hybrid-FA method was compared with the CWLS, NR, TSWLS, and GA methods using simulations and experiments. The RMSE of the hybrid-FA method was lower than that of the NR, TSWLS, and GA methods when the SNR ranged from 20 to 40 dB in the simulations. The result of the first experiment showed that the RMSE of the hybrid-FA method was 0.6778 m, which was lower than that of NR, TSWLS, and GA. The results of the second experiment illustrated that the mean distance error of the hybrid-FA method was 0.03419 m, which was lower than that of NR, TSWLS, and GA. On the whole, the hybrid-FA method outperformed the NR, TSWLS, and GA methods for TDoA measurement.

## Figures and Tables

**Table 1:** The root-mean-square error (RMSE) of the hybrid-FA and FA methods with different numbers of iterations.

	25 Iterations	30 Iterations	50 Iterations	100 Iterations
**FA method**	0.04334 m	0.04943 m	0.03762 m	0.03741 m
**Hybrid-FA method**	0.03744 m	0.03741 m	0.03741 m	0.03741 m

**Table 2:** The mean distance error of different methods.

Method	CWLS	Hybrid-FA	NR	TSWLS	GA
**Mean distance error (m)**	0.033205	0.03419	0.141656	0.062933	0.126473
